# Predictive Factors for Clinical Improvement Following a Manual Therapy-Based Program in Patients with Neck Pain: A Prescriptive Clinical Prediction Rule Derivation Study

**DOI:** 10.3390/reports9020098

**Published:** 2026-03-26

**Authors:** Emmanouil Kapernaros, Maria Moutzouri, Georgios Krekoukias, Nikolaos Chrysagis, George A. Koumantakis

**Affiliations:** 1 Master’s Degree Program “New Methods in Physiotherapy”, Physiotherapy Department, School of Health & Care Sciences, University of West Attica (UNIWA), 12243 Athens, Greece; moutzouri@uniwa.gr (M.M.); gkrekoukias@uniwa.gr (G.K.); nchrisagis@uniwa.gr (N.C.); gkoumantakis@uniwa.gr (G.A.K.); 2 Laboratory of Advanced Physiotherapy, Physiotherapy Department, School of Health & Care Sciences, University of West Attica (UNIWA), 12243 Athens, Greece

**Keywords:** cervical spine, clinical prediction rules, manipulative therapy, outcome

## Abstract

**Background**: The aim of this study was to derive and internally validate a prescriptive clinical prediction rule (CPR) for identifying baseline factors associated with short-term clinical improvement in patients with neck pain (NP) undergoing a manual therapy (MT)-based physiotherapy program. **Methods**: A prospective cohort study was conducted, including 71 patients with NP (18–65 years). Participants received six MT-based sessions over three weeks. Baseline assessments included Pain Intensity Numeric Rating Scale (PI-NRS), Neck Disability Index (NDI), Body Mass (BM), Body Mass Index (BMI), International Physical Activity Questionnaire-Short Form (IPAQ-SF), Hospital Anxiety and Depression Scale (HADS), Minnesota Satisfaction Questionnaire-Short Form (MSQ), and Craniovertebral Angle (CVA). Clinical improvement was defined using the Global Perceived Effect Scale (GPES-7). Univariate analyses, receiver operating characteristic (ROC) curve analysis, and forward stepwise logistic regression were performed to derive the predictive model. **Results**: Fifty-six participants (78.9%) reported moderate to complete improvement. BM ≥ 76.5 kg and MSQ score ≤ 42.5 were retained in the final regression model. When both predictors were present, the probability of clinical improvement increased to 96.43% (positive likelihood ratio = 7.58). The model demonstrated adequate fit (Nagelkerke R^2^ = 0.247; Hosmer–Lemeshow *p* = 0.804). Internal validation yielded an optimism-corrected AUC of 0.741, suggesting minimal overfitting. **Conclusions**: Higher BM and lower MSQ score were associated with greater short-term improvement following MT in patients with NP. These findings highlight the relevance of integrating physical and psychosocial factors in prescriptive rehabilitation approaches. External validation of this CPR is required before clinical implementation.

## 1. Background

Neck pain (NP) ranks as the fourth leading cause of disability worldwide, with a recurrence rate of approximately 50%, one year after its initial onset [1]. It presents with symptoms associated with multiple pathologies, with each patient having a unique presentation [2]. NP is categorized based on the duration of the symptoms (acute < 6 weeks, subacute 6 weeks–3 months, chronic > 3 months) [1], the mechanism of symptom onset (mechanical, nociceptive [1,3], non-specific [4], neuropathic [1], nociplastic [5], traumatic [4]), the location of the symptoms (centralized, non-centralized) [5,6,7], the severity (Grade 1–4) [8], and the recurrence pattern (single episode, recurrent, persistent) [8].

Various physical and demographic factors are associated with the development of NP, being more common in women compared to men [1,9,10], patients aged between 45 and 55 years [9], with a sedentary lifestyle [11,12], prolonged office or computer occupations, technical or healthcare professions [1], working for more than 6 h in a seated position [12,13], with previous neck injuries (traffic or sports trauma) [1], sleep disorders [14], increased BMI [13,15] or increased body mass (BM) [16]. Higher levels of physical activity (PA) provide protection [17] and prevention [13,18,19].

Psychological factors associated with NP include stress and psychological distress [20], anxiety [20], self-reported poor or moderate health perception [13], insufficient coping skills or strategies [9,21], kinesiphobia [21], catastrophizing [9], depression [21] and work-related factors (job stress, part-time employment, low satisfaction, unsupportive work environment) [1,12,15,20,22]. Additionally, socio-demographic factors such as smoking, increased family size, and low income [13] are linked to NP.

The variety of available treatments, combined with the limited and often conflicting evidence regarding their efficacy, presents significant challenges for healthcare professionals during the clinical reasoning process for managing NP [1]. By focusing on multiple factors that are predictive of a positive outcome of specific treatments while simultaneously matching patients’ goals, it not only improves their health outcomes, but it also reduces the socio-economic burden of NP through a patient-centered approach embedded within a biopsychosocial framework [23].

Manual therapy (MT) has been shown to reduce pain and improve functionality in patients with chronic, non-specific NP, compared to other interventions [24]. Manipulation techniques have demonstrated positive outcomes in managing chronic NP and disability [25], outperforming oral pharmaceutical treatments [26]. Moreover, the addition of exercise alongside mobilization techniques results in even better outcomes [27]. MT is believed to have three primary interaction mechanisms with the musculoskeletal system: (a) neurophysiological, (b) mechanical, and (c) psychological effects [28].

During the patient history and clinical evaluation, physiotherapists collect a wealth of information, which will be processed to design the optimal multimodal interventions, tailored to each patient [29]. Identifying subgroups of NP patients and categorizing them for rehabilitation based on their initial characteristics is recognized as a priority for improving management strategies [1,30]. Clinical prediction rules (CPRs) assist in categorizing patients according to their initial symptoms, enabling the selection of optimal clinical interventions based on the data obtained from the initial medical history and clinical evaluation [31,32].

CPRs can be classified as prognostic, identifying baseline factors associated with outcome within a given treatment, or prescriptive, identifying factors associated with the likelihood of successful response to a specific intervention [32,33]. The present study constitutes a prescriptive CPR derivation, identifying baseline characteristics associated with short-term improvement following an MT-based program.

The aim of this study was to develop and test the predictive ability of a prescriptive CPR in patients with NP based on the patients’ initial characteristics, following a six-session MT-based physiotherapy program over three weeks. The analysis was based on characteristics recorded before and after completing a physiotherapy program involving MT. Despite growing interest in prescriptive CPRs for neck pain, existing models have predominantly focused on biomechanical variables and have been derived in specific military or North American clinical settings, limiting their broader applicability. Furthermore, no prescriptive CPR has been developed for a multimodal MT-based program integrating psychosocial factors such as job satisfaction. This study aimed to address these gaps by developing a prescriptive CPR within a Greek clinical setting, contributing to the broader understanding of CPR applicability across different healthcare contexts.

## 2. Methods

### 2.1. Study Design

A prospective cohort study was conducted in a physiotherapy clinic located in the center of a major municipality in Attica, Greece. The study received approval from the Ethics Committee of the University of West Attica (UNIWA) (Approval No. 17778/11-03-2024), ensuring its design aligned with the principles of the Declaration of Helsinki.

### 2.2. Participants

Patients with NP (acute, subacute, or chronic stage) with centralized or non-centralized symptoms, aged 18–65, were included in the study. Patient history was taken according to IFOMPT guidelines [34] and previous studies that had administered MT in the cervical area [35,36]. Participants provided written informed consent before their inclusion. Complete anonymity was ensured, and all research data were encoded for statistical analysis.

The exclusion criteria used in this study are consistent with those applied in previous studies on MT for NP: (1) medical history of severe cervical pathology (osteoarthritis, rheumatoid arthritis, stenosis [10,37]); (2) non-musculoskeletal or non-psychological origin of symptoms; (3) history of cervical surgery or injury [38]; (4) osteoporosis [38]; (5) psychiatric disorders [38]; (6) history of tumors [10]; (7) pregnancy; (8) vascular; or (9) central nervous system pathologies [10,38]; (10) autoimmune diseases related to NP [38]; (11) inability to read and comprehend Greek [36]; (12) vertebrobasilar artery insufficiency or ligamentous instability of the upper cervical spine [10,38,39].

### 2.3. Patient-Reported Baseline Information

#### 2.3.1. International Physical Activity Questionnaire-Short Form (IPAQ-SF)

To calculate self-reported physical activity levels, the short form of the IPAQ-SF was used. It assesses the frequency and duration (in minutes per day) of activities performed for more than 10 min at vigorous intensity, moderate intensity, walking, and sedentary behavior over the past seven days [40]. The Greek version is a reliable and valid tool for assessing physical activity, with strong correlations to treadmill performance and significant inter-rater and test–retest reliability [41].

#### 2.3.2. Hospital Anxiety and Depression Scale (HADS)

HADS is a self-reported scale consisting of 14 items, with 7 questions each for assessing anxiety (HADS-A) and depression (HADS-D). Each item is scored on a 4-point scale, and the subscales yield scores ranging from 0 to 21, interpreted as: normal (0–7), mild anxiety/depression (8–10), moderate anxiety/depression (11–14), and severe anxiety/depression (15–21) [42]. It is translated and validated in Greek, shows strong internal consistency, test–retest reliability, structural validity, and high convergent validity with related measures [43].

#### 2.3.3. Minnesota Satisfaction Questionnaire (MSQ)—Short Form

The MSQ-short form is a 20-item questionnaire that measures job satisfaction on a 5-point scale, where 1 represents “very dissatisfied,” and 5 represents “very satisfied.” The total score, calculated by summing the item scores, ranges from 20 to 100, with higher scores indicating greater job satisfaction [44]. It is a valid, quick, and easy self-report tool for healthcare professionals [44,45].

#### 2.3.4. Baseline Disability

The Neck Disability Index (NDI) is a self-reported questionnaire comprising 10 items, each scored on a 6-point scale. The total score ranges from 0 to 50, with scores interpreted as follows: <4: no disability, 5–14: mild disability, 15–24: moderate disability, 25–34: severe disability, and >35: complete disability [46,47]. It is culturally adapted to Greek, reliably evaluates neck-related disability, and is sensitive to its severity and changes over time [46].

#### 2.3.5. Baseline Pain Intensity

The pain intensity numeric rating scale (PI-NRS) was used to assess the average intensity of NP over the past week. It consists of 11 options, where “0” represents “no pain” and “10” indicates “the worst possible pain.” A significant change in pain intensity is defined as a reduction of 1.5 to 4.1 points, depending on the intervention and clinical cause of NP [48]. It is a valid and reliable tool for measuring neck pain intensity, with high ease of use, reliability, validity, and responsiveness [48,49].

### 2.4. Subjective and Objective Patient Assessment

The subjective assessment included mechanisms, location, and nature of symptoms, symptom onset time, provocation and alleviation factors, previous musculoskeletal issues, and patient beliefs regarding potential NP improvement.

The clinical assessment involved neurological screening and evaluation of the mechanical properties of the region (Spurling test, distraction test, upper limb neurodynamic tests). Additional assessments included tests for cruciate and transverse ligament integrity in the upper cervical region and vertebrobasilar artery insufficiency [39].

#### Photogrammetry: Forward Head Posture (FHP) in Seated Work Position

The craniovertebral angle (CVA), derived from the forward head posture (FHP), was assessed using the PostureScreen Mobile^®^ application, version 13.7 (PostureCo Inc., Trinity, FL, USA) on a smartphone (iPhone, Apple Inc., Cupertino, CA, USA). The “Sit Screen” module was used by following a specific protocol from previous studies [50]. Although some disparity exists in the categorization of the range and severity of FHP, in the current study, CVA measurements were calculated from photographs of participants taken in simulated office work sitting positions, categorized as: normal (>53°), moderate FHP (52–46°), or severe deviation in FHP (<45°) [51]. This measure has previously demonstrated strong inter-rater and test–retest reliability [52].

Smaller CVA values indicate greater forward head displacement, which has been associated with increased mechanical load on the cervical spine, muscle imbalance, and higher risk of neck pain and disability [51,52]. The CVA has also demonstrated adequate criterion validity, with smaller angles correlating with greater forward head displacement as measured by gold-standard methods [52].

### 2.5. Outcome

#### Global Perceived Effect Scale (GPES-7)

Post-treatment clinical improvement was established according to the GPES-7. Patients completed the GPES-7 [53,54], a Likert scale frequently utilized in both research and clinical settings for musculoskeletal pathologies (Intraclass Correlation Coefficient (ICC) = 0.97), measuring clinical improvement during or after therapeutic interventions [53,54,55]. The GPES-7 has been cross-culturally adapted and validated in Greek, demonstrating excellent inter-rater reliability (*k* = 0.919, 95% Confidence Interval (CI): 83.3–92) [54].

### 2.6. Manual Therapy-Based Intervention

Patients included in the study received MT-based treatment using predefined methods tailored to each participant, based on a prior clinical assessment [35,38].

The intervention combined soft tissue techniques and mobilization (MT2), high velocity low amplitude thrust manipulation to the cervical and thoracic spine (MT1), and specific exercises, corresponding to an MT3 + exercise approach as defined by Hidalgo et al. [27].

The intervention protocol followed these steps [27]:

1. Soft Tissue Techniques: Performed in the prone or seated position, focusing on the cervical region (C1–T2), paraspinal areas, and muscles above the scapulae and clavicles for 5 min.
2. Passive and Active Movements: Passive neck movements (flexion, extension, lateral flexion, rotation, combined movements) targeting restricted directions, followed by active movements combined with mobilization techniques for 2 min each, in the supine or seated position.
3. Joint Techniques: Manipulations targeting restricted levels followed by mobilization for 2 min and traction at the disk level for 2 min, performed in the supine position.
4. Thoracic Spine Techniques: Manipulation techniques in the supine position were applied.
5. Rehabilitation Exercises: Isometric and posture retraining exercises in neutral and restricted positions (two contractions of 30 s each) were conducted in the supine and seated positions.
6. Stretching: Stretching of the scalene, levator scapulae, and upper trapezius muscles (one repetition for 30 s) was performed in the seated position.
7. Activities of Daily Living (ADL): Basic advice on ADL was provided, encouraging patients to maintain their usual activity levels if no symptom exacerbation occurred.

The entire intervention lasted 30–45 min. Although the intervention was standardized in structure, treatment techniques were adapted based on individual clinical findings, reflecting real-world physiotherapy practice.

Specifically, individualization was restricted to the anatomical level and side of dysfunction identified during clinical assessment (e.g., cervical level, direction of restriction), while the overall structure, sequence, and type of techniques remained identical across all participants. Patients were not consistently assigned to a single therapist; rather, treatment sessions were distributed among the four therapists based on appointment availability, which contributed to minimizing potential therapist-dependent effects. No systematic recording of the specific techniques applied per participant per session was performed.

The treatment was administered by the principal investigator with over 17 years of professional experience, and three physiotherapists, each with at least four years of experience in MT. Therapists were trained to apply only the specified treatment methods without being informed of the study’s purpose.

### 2.7. Procedures

Data were collected through clinical examinations, patient self-reports using validated Greek versions of the questionnaires, and photogrammetry conducted in a setting where only the patient and the examiner were present.

Assessments were conducted at two time points: (1) baseline (pre-treatment), during which all demographic data, patient-reported outcome measures (PI-NRS, NDI, IPAQ-SF, HADS, MSQ), and photogrammetry (CVA) were collected; and (2) post-treatment (following the sixth session, approximately after three weeks from the baseline measurement), during which PI-NRS, NDI, and GPES-7 were re-administered to evaluate changes and determine clinical improvement.

The selection of predictive factors (demographics, assessment data, and self-reported questionnaires) and outcome followed similar cohort studies aimed at developing CPRs for NP [56,57,58,59,60]. Self-reported questionnaire-based measures were predominantly selected, as they are considered valid prognostic tools for investigating improvement outcomes in patients with NP [61]. Sample size restrictions guided the optimal selection of patient characteristics for CPR development [62,63].

After the six treatments, repeat measurements were conducted to evaluate changes in pain intensity (PI-NRS) and disability (NDI), and patient satisfaction (GPES-7) [36,58,64].

### 2.8. Sample Size

The sample size calculation followed the empirical ratio of 1:10 for the required number of subjects per variable [36,62,63,64]. Although seven baseline variables were initially considered, only three met the univariate screening criterion, and two were retained in the final model.

### 2.9. Statistical Analysis

Statistical analysis was performed with IBM Statistical Package for the Social Sciences (SPSS), version 29 (IBM Corp., Armonk, NY, USA) [65], MedCalc (MedCalc’s Diagnostic Test Evaluation Calculator) [66], and R statistical software (v.4.5.3; R Core Team, 2025) [67]. Bootstrap validation of regression coefficients was performed in SPSS, and optimism-corrected AUC was estimated using the rms package in R [68].

#### 2.9.1. Normality Testing

The Shapiro–Wilk test was applied to assess the normal distribution of quantitative variables [69]. The GPES-7 was used exclusively for dichotomization into ‘improved’ and ‘not improved’ groups; no parametric operations were applied to its raw scores, and no assumption of equal intervals was required [36,64]. Separate analysis was conducted for IPAQ-SF data due to missing responses (*n* = 15, 21.1% of the sample) [41]. Missing responses arose from items not meeting scoring criteria (activities <10 min, >180 min/day, or ‘don’t know’ responses) and were handled using complete-case analysis per standard IPAQ scoring guidelines. To assess whether missingness was associated with the primary outcome, a chi-square analysis comparing improvement rates between participants with and without complete IPAQ-SF data yielded no statistically significant association (χ^2^ = 1.370, df = 2, *p* = 0.504), supporting the assumption of missing at random.

Continuous numerical variables were presented as means ± standard deviations or medians (interquartile ranges), depending on whether they followed a normal distribution, while categorical variables were expressed as percentages.

#### 2.9.2. Identifying Predictive Factors for MT-Based Intervention

Post-intervention data were dichotomized into two groups based on GPES-7 scores. Patients reporting scores of 1 (“fully recovered”) or 2 (“considerably improved”) were classified as improved. All other responses (scores ≥ 3) were classified as not improved [36,70,71].

The mean change in scores for pain and disability (including interquartile ranges) was calculated for both groups and analyzed using the Mann–Whitney U test to confirm differences between groups, as determined by GPES-7.

##### Univariate Analysis

Univariate analysis was performed using independent sample *t*-tests or Mann–Whitney U tests for continuous variables and chi-square tests (χ^2^) for categorical variables. Continuous variables with a significance level of *p* ≤ 0.1 were included in subsequent multivariate analysis to minimize the likelihood of excluding potential predictive factors [36,38,72,73,74].

##### Receiver Operating Characteristic (ROC) Curve Analysis

For continuous variables that showed significant univariate relationships, sensitivity and specificity were calculated for all potential cutoff points, and ROC curves were plotted [36,75,76]. The area under the curve (AUC) was computed to assess accuracy. The Youden Index was used to determine the optimal cutoff point for defining a positive test [77].

Confidence intervals and likelihood ratios were calculated using MedCalc, with continuity correction applied by adding 0.5 to cells with a value of zero [78].

##### Selection of Predictive Factors

Potential predictive variables identified through univariate analysis were entered into a forward stepwise binary logistic regression model to identify the most accurate and parsimonious set of variables for predicting the success of MT in improving clinical outcomes for NP patients [64]. A *p*-value < 0.05 was required for inclusion in the model, and variables with *p* > 0.10 were excluded.

The goodness of fit for the final regression model was assessed using the Hosmer-Lemeshow statistic [79]. The proportion of variance explained by the model was determined using Nagelkerke R^2^ [80]. The final model retained two predictors, yielding approximately 28 events per variable, which exceeded the commonly recommended threshold of 10, reducing the likelihood of model overfitting.

To further assess model stability, two internal validation procedures were performed: (a) bootstrap validation of regression coefficients using 1000 bootstrap samples with bias-corrected and accelerated (BCa) 95% confidence intervals (SPSS v.29), and (b) optimism-corrected c-statistic (AUC) using 1000 bootstrap samples via the validate() function of the rms package in R.

## 3. Results

Between February and August 2024, 83 patients were approached for study inclusion. Of these, 12 were excluded due to various reasons (rheumatic disease, refusal to participate, incomplete sessions), leaving a final sample of 71 participants (52 females).

All participants completed the questionnaires and photogrammetry measurements fully, except for some responses in the IPAQ-SF involving activities under 10 min or over 180 min per day, undefined durations, or “don’t know/not sure” selections [41].

Among the 71 patients, 56 (78.9%) reported improvement, while 15 (21.1%) did not. No patients reported worsening symptoms. The Mann–Whitney U tests revealed statistically significant differences in pain and disability improvement scores between the improved and non-improved groups, as defined by the GPES-7 (Table 1).

The descriptive statistics for the 71 participants and the univariate relationships of the 12 variables with the GPES-7 criteria were presented in Table 2. Continuous numerical variables were expressed as means ± standard deviations or medians (interquartile ranges), while categorical variables were expressed as percentages.

Univariate analysis identified BM, BMI, initial pain intensity (PI-NRS), and job satisfaction (MSQ) as predictive variables for improvement. However, BMI was excluded to avoid multicollinearity with BM. Consequently, three variables were retained for the final regression model.

### 3.1. ROC Curve Analysis

**Figure 1:**
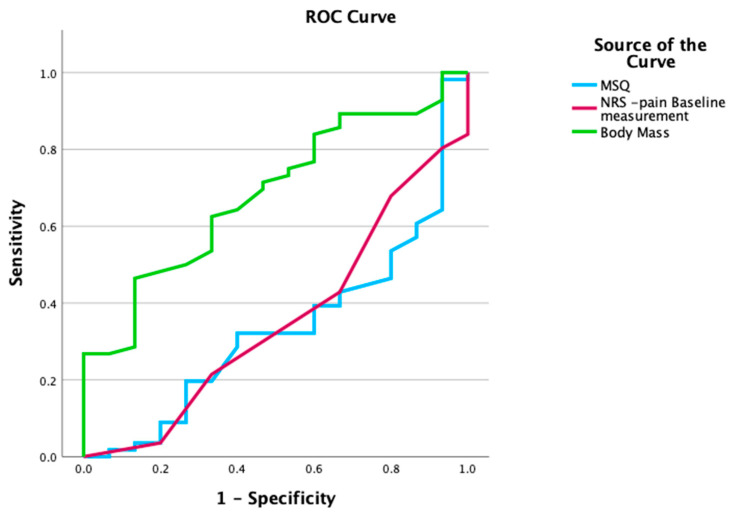
ROC curves for the three variables with statistically significant univariate association with clinical improvement: BM, PI-NRS, and MSQ. The AUC reflects the discriminative ability of each variable, with optimal cut-off points determined using the Youden Index. For PI-NRS and MSQ, 1-AUC was applied due to the reversed direction of prediction.

ROC curves were plotted for the three potential predictive factors to determine the cutoff points for defining positive tests as presented in Figure 1. The results of the statistical accuracy analysis for all three variables are summarized in Table 3.

### 3.2. Selection of Predictive Factors

The final binary logistic regression model, based on forward stepwise inclusion of variables, retained the variables BM ≥ 76.5 kg and MSQ ≤ 42.5. The statistical analysis of the model yielded a χ^2^ = 12.297 with df = 2 and *p* = 0.002, indicating that the model is statistically significant. The Nagelkerke R^2^ value of 0.247 suggests that the model explains approximately 24.7% of the variance in the dependent variable and demonstrated acceptable fit, as shown by the Hosmer–Lemeshow test χ^2^ = 0.437, *p* = 0.804, indicating that the model’s predictions align well with the observed data. The regression coefficients and accuracy statistics for the final model are presented in Table 4.

Bootstrap validation of regression coefficients (1000 BCa samples) confirmed the statistical significance of both retained predictors: BM ≥ 76.5 kg (B = 1.855, *p* = 0.013, BCa 95% CI: 0.078–21.187) and MSQ ≤ 42.5 (B = 1.653, *p* = 0.011, BCa 95% CI: −0.113–21.286). Optimism-corrected internal validation yielded an apparent AUC of 0.759, with an optimism estimate of 0.035 and an optimism-corrected AUC of 0.741, indicating minimal overfitting.

To further evaluate the association between BM and short-term improvement, baseline NDI and PI-NRS scores were compared between participants with BM ≥ 76.5 kg and those with BM < 76.5 kg using independent samples *t*-tests. No statistically significant differences were found (NDI: 13.04 ± 9.18 vs. 13.02 ± 6.22, *p* = 0.995; PI-NRS: 6.93 ± 1.68 vs. 7.23 ± 2.08, *p* = 0.519), suggesting that baseline symptom severity does not differ between BM groups.

The observed improvement rate (78.9%) was used as the pre-test probability to calculate post-test probabilities. Positive likelihood ratios and post-test probabilities were calculated for each level of the prediction model. The diagnostic performance of the MT success model is presented in Table 5. Diagnostic accuracy indices in Table 5 were calculated based on the logistic regression model classification table rather than the ROC-derived single-variable performance.

## 4. Discussion

The purpose of this study was to identify a subgroup of patients with neck pain (NP) based on their initial characteristics [29,32,81], who would exhibit short-term improvement following the application of MT, aiming to improve management strategies [1,30]. In physiotherapy, related studies are primarily conducted to develop prescriptive CPRs for musculoskeletal problems, by collecting factors from subjective and objective assessment [81], by studying their predictive value on clinical improvement in patients with a specific pathology and selected rehabilitation interventions [32,33,81].

A prescriptive CPR identifying baseline characteristics associated with clinical improvement may assist in targeted rehabilitation planning.

The identified factors predictive of improvement with MT were BM ≥ 76.5 kg and MSQ ≤ 42.5. The use of stepwise selection may increase the risk of overfitting and should therefore be interpreted cautiously. However, two internal validation procedures were subsequently performed: bootstrap validation of regression coefficients (1000 BCa samples) and optimism-corrected AUC estimation (1000 bootstrap samples). The optimism estimate was small (0.035), with an apparent AUC of 0.759 corrected to 0.741, suggesting minimal overfitting. Nevertheless, the derived model should be considered exploratory and hypothesis-generating, and external validation remains necessary.

When the 1-variable model (MSQ ≤ 42.5) was positive, sensitivity was 53.6%, and the positive likelihood ratio was 1.61, increasing the post-test probability of improvement to 85.71%. When both predictors were present (BM ≥ 76.5 kg and MSQ ≤ 42.5), specificity increased to 96.9%, the positive likelihood ratio increased to 7.58, and the post-test probability of improvement increased to 96.43%.

The GPES-7 was selected to dichotomize the sample because it reflects patients’ overall perception of improvement, rather than solely pain or disability measurements [54]. To avoid overestimating minor improvements, only patients reporting at least “considerable improvement” were classified as “improved” [64].

Increased BM could possibly be associated with neck pain, due to higher systemic inflammation, structural changes, mechanical loads, and reduced muscle strength [16]. Additionally, psychosocial issues and greater disability, including kinesiophobia, are prevalent in overweight and obese individuals [16]. Most patients in the study were overweight or obese (*n* = 41, 57.7%, with a mean BM of 75.38 kg). Although regression to the mean was considered as a possible explanation, given that individuals with higher BM may present with greater baseline impairment, comparison of baseline NDI and PI-NRS scores between BM groups revealed no statistically significant differences (NDI: *p* = 0.995; PI-NRS: *p* = 0.519). These findings do not support baseline severity as the primary explanatory mechanism. An alternative hypothesis is that higher BM may be associated with greater mechanical loading of the cervical spine, potentially rendering these patients more responsive to the biomechanical effects of MT [16]. However, the underlying mechanisms remain speculative, and formal mediation or interaction analysis is recommended in future studies with larger samples.

Workplace conditions may expose employees to various psychological factors that can significantly disrupt their mental and physical health [82]. Work-related stress, part-time employment, and low job satisfaction are recognized as contributors to the development of neck pain [12,20], as well as disability [1,15,22]. It is possible that patients with lower job satisfaction experience greater perceived improvement due to contextual or expectancy-related factors. Furthermore, job satisfaction has been identified as a significant independent predictor of musculoskeletal pain outcomes, beyond the variance explained by baseline pain and disability [83]. The causal pathways underlying this association require further investigation, and formal mediation analysis is recommended in future studies.

A marginal correlation was observed between PA and improvement following the application of MT. PA is expected to positively influence pain and disability and should likely result in statistically significant better outcomes in the rehabilitation of patients with NP [13,17,18,19]. This finding could stem from improper completion of the questionnaire by patients or the questionnaire’s inability to identify those aspects of PA that positively impact the cervical region [19,84,85,86].

Psychological factors (depression, anxiety) and the CVA did not predict improvement in NP patients following MT. These findings align with other studies [38,87]. Similarly, initial pain intensity was not selected, potentially due to a higher mean pain level in the current study [23], but also when compared to related studies [58,59,64,88].

MT encompasses a wide array of methods and approaches for addressing musculoskeletal pathologies. The type of intervention should be clearly defined to investigate the impact of each methodology on a specific population in each study [2,25,27].

Nine studies were identified in the literature investigating MT-based interventions for NP, aimed at developing a prescriptive CPR [35,36,38,56,57,58,59,60,64]. They demonstrate that identifying prognostic factors, including symptom duration, cervical range of motion, and pain intensity, may effectively guide treatment strategies [36,56,57]. Specifically, the use of combined criteria, such as positive responses to specific tests and pain severity, can accurately predict the success of interventions like thoracic and cervical mobilization [35,57] or mechanical cervical traction [64]. However, the heterogeneity in study populations, the absence of control groups, and small sample sizes limit the generalizability of these findings, as noted in related research [36,60]. Despite these limitations, these findings underscore the importance of well-designed predictive models in improving clinical decision-making and the effectiveness of interventions.

Unlike studies that emphasized biomechanical variables such as cervical range of motion [59,64], this study underscores the interplay of psychosocial elements, including job satisfaction, furthering the evidence for integrating a biopsychosocial framework in MT applications. Despite methodological differences, the findings collectively suggest that the potential value of personalized rehabilitation strategies, emphasizing the inclusion of both physical and psychological factors, lies in optimizing clinical outcomes in neck pain management.

Regarding the external validation of prescriptive CPRs for NP with MT-based therapy applications, just one study was found in the literature [89]; CPRs in primary healthcare, particularly for NP, often remain at the derivation stage and lack comprehensive validation and impact analysis, limiting their routine clinical applicability [33]. Medical personnel often exhibit a prevailing fear of missed diagnoses, reliance on personal clinical judgment, and sensitivity to patients’ beliefs, favoring further investigation and management of their condition, ultimately leading to the suboptimal use of CPRs [90].

Although the present prescriptive CPR is not yet validated for widespread clinical use, it provides a valuable framework for identifying potential predictive factors. Future studies should focus on its external validation to confirm its applicability and improve decision-making in physiotherapy practice. Furthermore, clinical trials with control groups are recommended over prospective cohort studies. This would allow a clearer examination of patient improvement due to the intervention being tested in relation to predictive factors [33].

Future CPR development studies should avoid convenience sampling to ensure results are robust and can be validated in subsequent studies. Additionally, these studies should adhere to strict external validity criteria and favor randomized controlled trial designs over prospective cohort studies to confirm that observed outcomes result from the interventions under investigation.

A notable limitation of this study is the reliance on a convenience sampling method, which may introduce response bias [91] and the influence of social desirability effects [92,93]. Additionally, the relatively small sample size restricts the generalizability of the findings. Although internal validation—including bootstrap confidence intervals and optimism-corrected AUC (corrected AUC = 0.741)—suggested minimal overfitting, the wide bootstrap confidence intervals for both predictors indicate estimation imprecision that warrants cautious interpretation. Furthermore, the lack of a control group means that the identified predictors cannot be confirmed as true treatment-effect modifiers specific to MT, as opposed to non-specific prognostic factors for improvement regardless of intervention. This is a recognized methodological limitation of single-arm prescriptive CPR derivation studies [33], and external validation using a randomized controlled design with an active comparator is required to establish the prescriptive validity of this rule.

Additionally, the specific manual therapy techniques applied to each participant were not systematically recorded per session, limiting the ability to examine technique-level effects. However, it should be noted that individualization of treatment was restricted to the anatomical level and side of dysfunction, while the overall structure and type of techniques remained standardized across all participants. Furthermore, potential therapist clustering effects were mitigated by the rotating assignment of patients among the four therapists throughout the study, which also allowed the principal investigator to observe treatment delivery indirectly. Formal clustering analysis was not performed, and future studies should consider multilevel modeling to account for potential therapist-dependent variability.

The emergence of BM and job satisfaction as predictors of short-term improvement following MT highlights the potential relevance of physical and psychosocial baseline characteristics in shaping treatment response. These findings support the clinical value of a biopsychosocial assessment approach, suggesting that factors beyond pain intensity and disability, such as occupational psychosocial burden, may influence patients’ responsiveness to MT-based interventions. While these observations require external validation before informing clinical decision-making, they underscore the importance of integrating both physical and psychosocial domains into the assessment and planning of MT-based rehabilitation for NP.

This study represents the derivation phase of a prescriptive CPR and should not be applied in routine clinical decision-making without external validation.

## 5. Conclusions

NP is a multifactorial condition, and rehabilitation programs should equally address physical and psychological factors. The reciprocal relationship between neck pain and disability underscores the need for a comprehensive approach.

Internal validation procedures, including bootstrap coefficient estimation and optimism-corrected AUC (0.741), provided preliminary evidence of model stability, supporting the exploratory value of these findings pending external validation.

BM and job satisfaction may play a crucial role in the rehabilitation of NP patients through MT-based interventions. This study adds to the growing evidence supporting the need for tailored rehabilitation approaches in NP patients, emphasizing the role of demographic and psychosocial factors. Future research should explore the integration of these factors in multidisciplinary care models.

## Figures and Tables

**Table 1:** Patient-reported clinical improvement GPES-7 (*n* = 71).

Patient Perception	*n* (%)	Dichotomization *n* (%)	PI-NRS Change	NDI Change
Fully recovered Considerable improvement Slight improvement No change	17 (23.9%) 39 (54.9%) 12 (16.9%) 3 (4.2%)	56 (78.9%) Improved	3.73 (3–5) *p* < 0.001	6.58 (3–10) *p* = 0.007
		15 (21.1%) Not improved		

**Table 2:** Univariate analysis comparisons of demographic data, self-report clinical status questionnaires, and photogrammetry between improved and non-improved patients according to the GPES-7 scale change.

Continuous Variables				
Characteristic	All Participants (*n* = 71)	Improved Participants *n* = 56 (78.9%)	Non-Improved Participants *n* = 15 (21.1%)	GPES-7 Based Univariate Analysis (*p*-Values)
Age (years)	42.66 (34–51)	43.14 (35.25–51.75)	40.87 (31–50)	0.535
Height (m)	1.68 (1.62–1.75)	1.69 (1.63–1.75)	1.67 (1.60–1.74)	0.467
BM (kg)	75.38 (±18.35)	77.84 (±18.9)	66.2 (±12.76)	**0.028**
BMI (kg/m^2^)	26.26 (22.14–28.4)	27.02 (23.91–29.38)	23.42 (21.48–26.5)	**0.032**
PI-NRS Baseline	7.11 (6–8)	6.89 (6–8)	7.93 (7–9)	**0.081**
PI-NRS Final		3 (2–4)	6 (5–7)	
PI-NRS Change		4.16 (3–5)	2.13 (1–3)	
NDI Baseline	13.03 (8–17)	12.93 (8–17)	13.4 (9–15)	0.606
NDI Final		5.48 (3–8)	10.07 (8–13)	
NDI Change		7.45 (3–10.75)	3.33 (−1–6)	
HADS Anxiety	7.87 (±3.71)	8.04 (±3.72)	7.27 (±3.71)	0.479
HADS Depression	6.07 (±3.34)	6.38 (±3.37)	4.93 (±3.11)	0.139
HADS Total	13.94 (±6.12)	14.41 (±6.06)	12.2 (±6.22)	0.216
MSQ	44.93 (±15)	43.13 (±14.56)	51.67 (±15.23)	**0.049**
CVA	42.29 (±9.99)	42.38 (±9.67)	41.92 (±11.46)	0.877
IPAQ-SF MET/week	2248.23 (675–2860.25)	2431.47 (720–2916)	1585.69 (306–2921.5)	0.247
IPAQ-SF sitting h/day	7.29 (4–10)	7.04 (4–10)	8.31 (3.5–12)	0.310
Gender Men Women	19 (26.8%) 52 (73.2%)	16 (28.6%) 40 (71.4%)	3 (20%) 12 (80%)	0.505
Age Category 20 to 30 30 to 40 40 to 50 50 to 60 >60	10 (14.1%) 21 (29.6%) 19 (26.8%) 11 (15.5%) 10 (14.1%)	8 (14.3%) 16 (28.6%) 15 (26.8%) 8 (14.3%) 9 (16.1%)	2 (13.3%) 5 (33.3%) 4 (26.7%) 3 (20%) 1 (6.7%)	
BMI (kg/m^2^) Categories Underweight Normal Overweight Obese	3 (4.2%) 27 (38%) 28 (39.4%) 13 (18.3%)	2 (3.6%) 19 (33.9%) 22 (39.3%) 13 (23.2%)	1 (6.7%) 8 (53.3%) 6 (40%) -	
Chronicity (months) Acute–Subacute Chronic	4 (2–7) 30 (42.3%) 41 (57.7%)	6.07 (2–6) 26 (46.4%) 30 (53.6%)	8.6 (2–12) 4 (26.7%) 11 (73.3%)	0.182
Office occupation Yes No	42 (59.2%) 29 (40.8%)	33 (58.9%) 23 (41.1%)	9 (60%) 6 (40%)	0.940
Symptom localization Centralized Non centralized	44 (62%) 27 (38%)	36 (64.3%) 20 (35.7%)	8 (53.3%) 7 (46.7%)	0.438
IPAQ-SF Low Moderate High	26 (36.6%) 28 (39.4%) 17 (23.9%)	19 (33.9%) 24 (42.9%) 13 (23.2%)	7 (46.7%) 4 (26.7%) 4 (26.7%)	0.504
NDI Baseline No Mild Moderate Severe Complete	3 (4.2%) 44 (62%) 17 (23.9%) 6 (8.5%) 1 (1.4%)	3 (5.4%) 33 (58.9%) 15 (26.8%) 4 (7.1%) 1 (1.8%)	11 (73.3%) 2 (13.3%) 3 (13.3%) - -	
NDI Final No Mild Moderate Severe Complete		31 (55.4%) 22 (39.3%) 2 (3.6%) 1 (1.8%) -	2 (13.3%) 11 (73.3%) 2 (13.3%) - -	
HADS Anxiety Normal Mild Moderate Severe	34 (47.9%) 25 (35.2%) 10 (14.1%) 2 (2.8%)	25 (44.6%) 21 (37.5%) 8 (14.3%) 2 (3.6%)	9 (60%) 4 (26.7%) 2 (13.3%) -	
HADS Depression Normal Mild Moderate Severe	49 (69%) 14 (19.7%) 7 (9.9%) 1 (1.4%)	37 (66.1%) 12 (21.4%) 6 (10.7%) 1 (1.8%)	12 (80%) 2 (13.3%) 1 (6.7%) -	
MSQ Employee Self-employed	63 (88.7%) 8 (11.3%)	48 (85.7%) 8 (14.3%)	15 (100%) -	
CVA Normal FHP Severe deviation	6 (8.5%) 28 (39.4%) 37 (52.1%)	5 (8.9%) 21 (37.5%) 30 (53.6%)	1 (6.7%) 7 (46.7%) 7 (46.7%)	

Bold values indicate *p* ≤ 0.1 (threshold for inclusion in multivariate analysis).

**Table 3:** Sensitivity, specificity, and likelihood ratios for the variables with statistically significant univariate relationships with the improvement from MT interventions. For PI-NRS and MSQ values, 1-AUC was applied due to the reversed prediction model.

Variable	Value	Sensitivity	Specificity	Positive Likelihood Ratio	Negative Likelihood Ratio	AUC	*p*-Value	95% CI
BM	≥76.5	0.867 (0.595–0.984)	0.464 (0.323–0.603)	1.62 (1.18–2.22)	0.29 (0.08–1.08)	0.683	0.010	0.543–0.823
PI-NRS Baseline	≤7.5	0.667 (0.384–0.882)	0.571 (0.432–0.703)	1.56 (0.97–2.49)	0.58 (0.28–1.24)	0.645	0.067	0.490–0.800
MSQ	≤42.5	0.800 (0.519–0.957)	0.536 (0.397–0.670)	1.72 (1.18–2.52)	0.37 (0.13–1.06)	0.667	0.029	0.517–0.818

**Table 4:** Binary logistic regression model coefficients for the final two-variable prescriptive CPR.

Variable	B	SE	Wald	df	*p*-Value	OR	95% CI
BM ≥ 76.5 kg	−1.855	0.829	5.004	1	0.025	0.156	0.031–0.795
MSQ ≤ 42.5	−1.653	0.725	5.203	1	0.023	0.191	0.046–0.792
Constant	−0.208	0.411	0.256	1	0.613	0.812	

The outcome variable was clinical improvement as defined by GPES-7 dichotomization. A positive GPES-7 outcome (improvement) was coded as 0, and a non-improvement as 1; therefore, negative B values indicate association with a greater likelihood of improvement.

**Table 5:** Comparison of sensitivity, specificity, and likelihood ratios for the 1-variable and 2-variable models of the prescriptive CPR developed in this study.

Number of Predictive Variables	Sensitivity	Specificity	Positive Likelihood Ratio	Overall Proportion Correctly Classified	Probability of Success by Applying MT (PPV)	Improved Patients	Non-Improved Patients
1 variable *	0.536 (0.397–0.670)	0.667 (0.384–0.882)	1.61 (0.75–3.42)	56.34% (44.05–68.09%)	85.71% (73.80–92.74%)	30	5
Both variables	0.237 (0.202–0.273)	0.969 (0.929–0.99)	7.58 (3.16–18.19)	39.73% (36.16–43.38%) **	96.43% (91.84–98.48%)	13	0

In the analysis of both characteristics, 0.5 was added to the initial count of each cell where “0” appeared in the “no improvement and absence of characteristic”. * The 1-variable model corresponds to MSQ ≤ 42.5, as it demonstrated the strongest independent predictive performance in the univariate and ROC analyses. ** The low overall accuracy reflects the low sensitivity of the combined rule, which was designed to maximize specificity and positive likelihood ratio rather than overall classification performance.

## Data Availability

The data presented in this study are available from the corresponding author upon reasonable request. The data are not publicly available due to privacy and ethical restrictions.

## Abbreviations

The following abbreviations are used in this manuscript:

NP	Neck Pain
CPR	Clinical Prediction Rule
PI-NRS	Pain Intensity Numeric Rating Scale
NDI	Neck Disability Index
BM	Body Mass
BMI	Body Mass Index
IPAQ-SF	International Physical Activity Questionnaire-Short Form
HADS	Hospital Anxiety and Depression Scale
MSQ	Minnesota Satisfaction Questionnaire-Short Form
CVA	Craniovertebral Angle
GPES-7	Global Perceived Effect Scale
PA	Physical Activity
MT	Manual Therapy
IFOMPT	International Federation of Orthopaedic Manual Therapy
FHP	Forward Head Posture
ICC	Intraclass Correlation Coefficient
CI	Confidence Interval
ADL	Activities of Daily Living
SPSS	Statistical Package for the Social Sciences
ROC	Receiver Operating Characteristic
AUC	Area Under the Curve
PPV	Positive Predictive Value
MET	Metabolic Equivalent
UNIWA	University of West Attica
B	Unstandardized Regression Coefficient
SE	Standard Error
OR	Odds Ratio
